# Supplementary material for: An Optimized Screen Reduces the Number of GA Transporters and Provides Insights Into Nitrate Transporter 1/Peptide Transporter Family Substrate Determinants
Source: Front Plant Sci. 2019 Oct 3;10:1106. doi: 10.3389/fpls.2019.01106 (PMC6785635; doi:10.3389/fpls.2019.01106)
Supplement: Supplementary file 1 [file Table_1.docx]

Supplementary Material


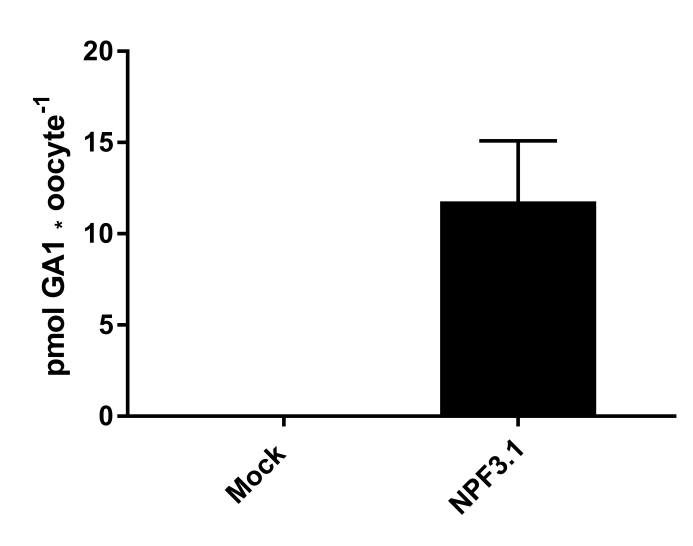

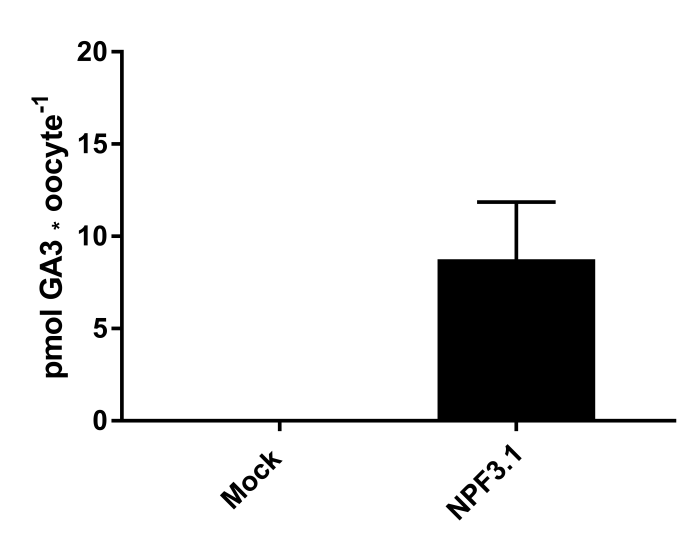

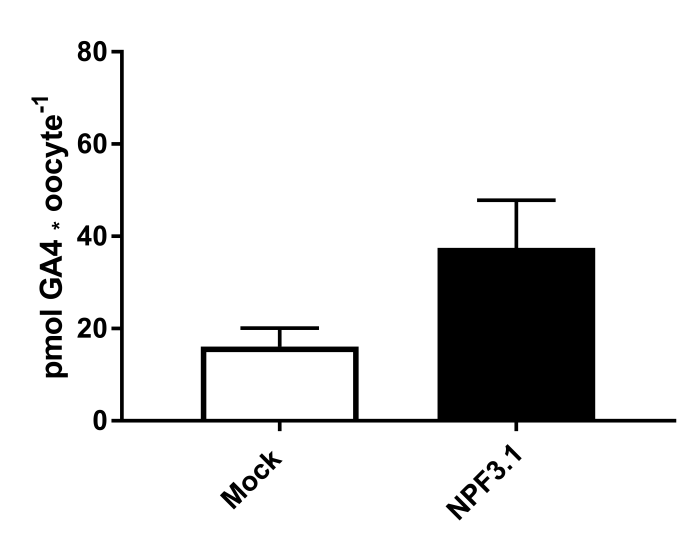

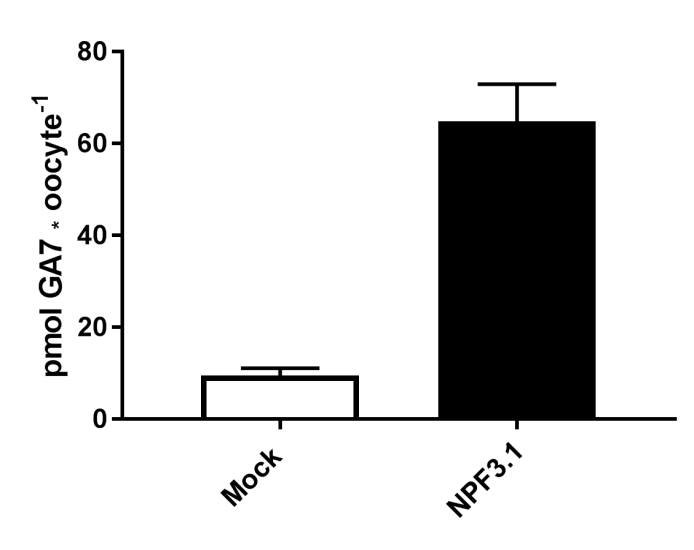

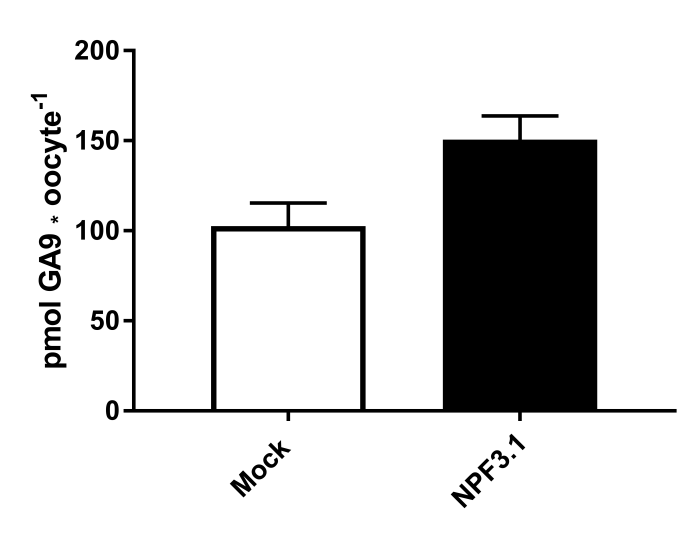

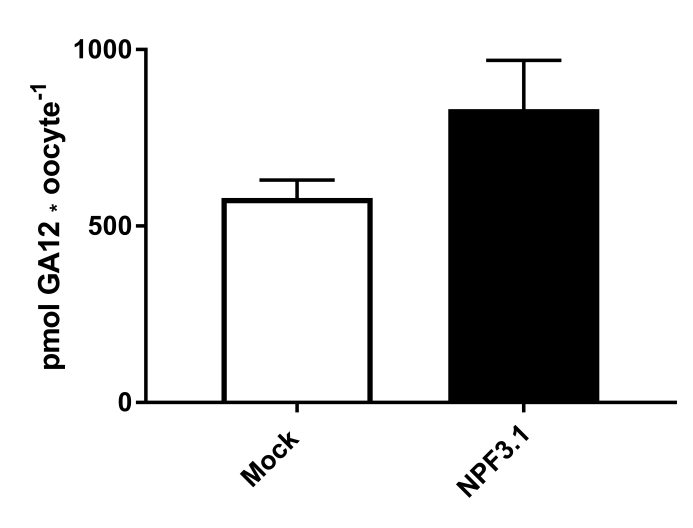

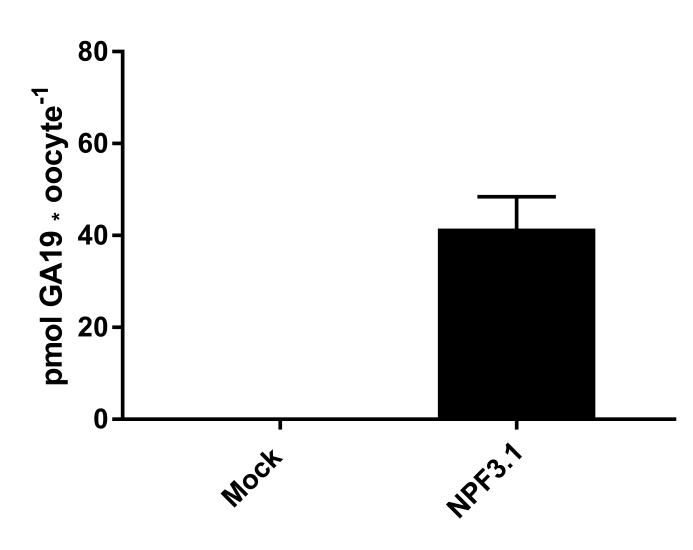

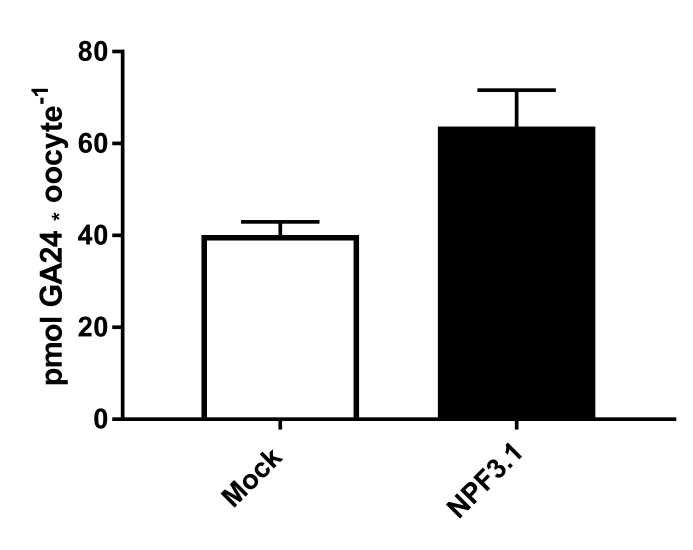


**Supplementary Figure 1.** Expanded list of NPF3.1 GA substrates. Membrane non-permeable GAs (GA1, GA3 and GA19) were assayed in MES based kulori pH 5 for 1 h. Moderately membrane permeable GAs (GA4, GA7 and GA24) were assayed in MES based kulori pH 5.5 for 1 h. Highly membrane permeable GAs (GA9 and GA12) were assayed in MES based kulori pH 6 for 1 h. All transport events are statistically significant (Holm Sidak one-way ANOVA p = 0.05).
