# Supplementary material for: An Optimized Screen Reduces the Number of GA Transporters and Provides Insights Into Nitrate Transporter 1/Peptide Transporter Family Substrate Determinants
Source: Front Plant Sci. 2019 Oct 3;10:1106. doi: 10.3389/fpls.2019.01106 (PMC6785635; doi:10.3389/fpls.2019.01106)
Supplement: Supplementary file 2 [file Table_2.docx]

Supplementary Material


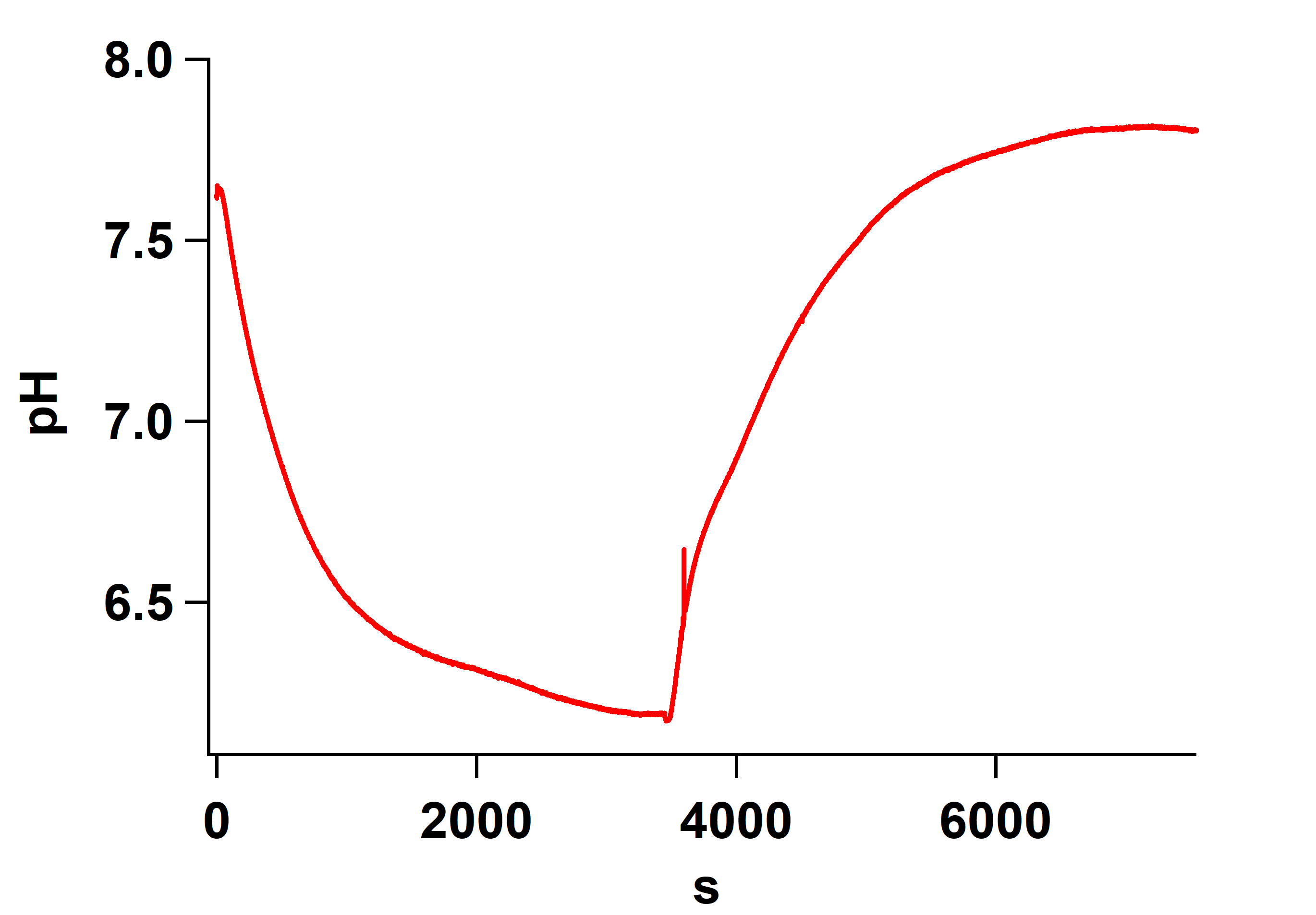


**Supplementary Figure 2.** Cytosolic pH of NPF7.3 expressing oocytes upon exposure to pH 5.0 for 60 mins followed by 60 mins in pH 7.4
