# Supplementary material for: An Optimized Screen Reduces the Number of GA Transporters and Provides Insights Into Nitrate Transporter 1/Peptide Transporter Family Substrate Determinants
Source: Front Plant Sci. 2019 Oct 3;10:1106. doi: 10.3389/fpls.2019.01106 (PMC6785635; doi:10.3389/fpls.2019.01106)
Supplement: Supplementary file 3 [file Table_3.docx]

Supplementary Material


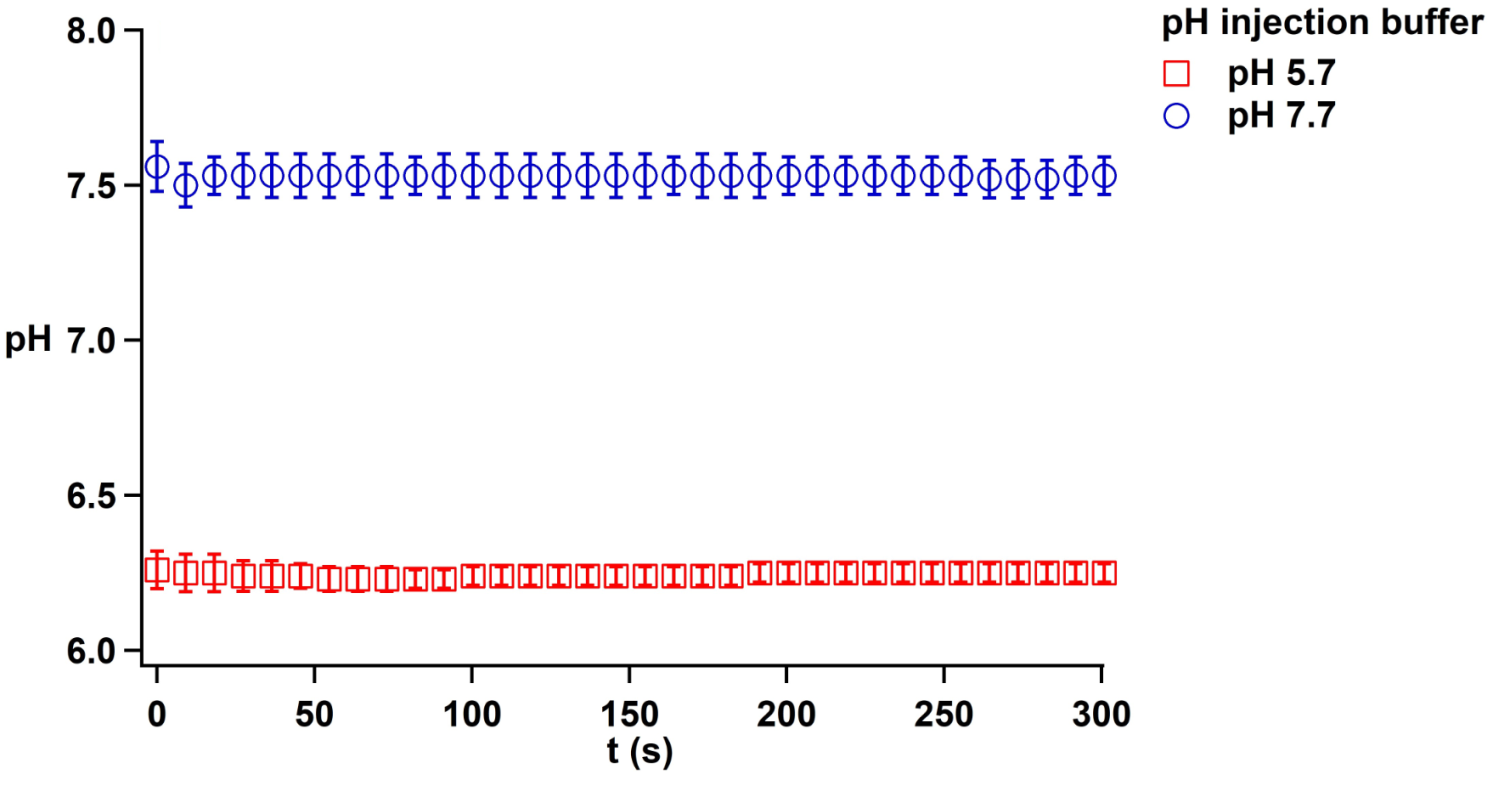


**Supplementary Figure 3.** Internal pH of oocytes injected with 50.6 nl 0.5 M TRIS 50 mM EGTA adjusted to pH 7.7 with 0.5 M MES (blue circles) and internal pH of oocytes injected with 50.6 nl 0.5 M MES 50 mM EGTA adjusted to 5.7 with 0.5 M TRIS (red squares).
