# Supplementary material for: An Optimized Screen Reduces the Number of GA Transporters and Provides Insights Into Nitrate Transporter 1/Peptide Transporter Family Substrate Determinants
Source: Front Plant Sci. 2019 Oct 3;10:1106. doi: 10.3389/fpls.2019.01106 (PMC6785635; doi:10.3389/fpls.2019.01106)
Supplement: Supplementary file 4 [file Table_4.docx]

Supplementary Material


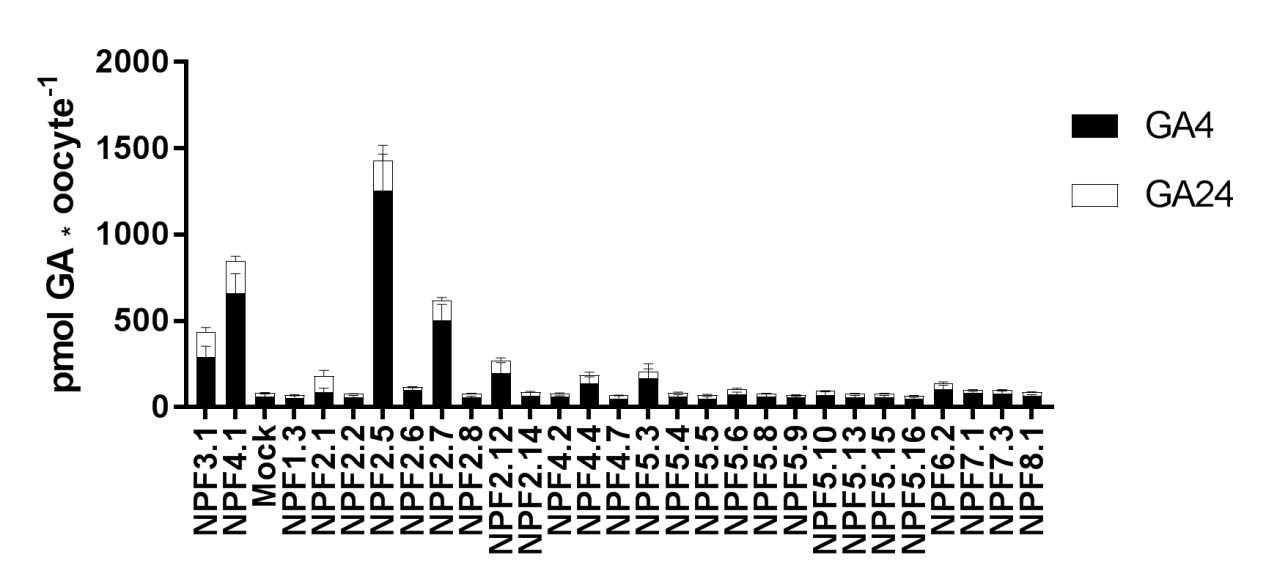

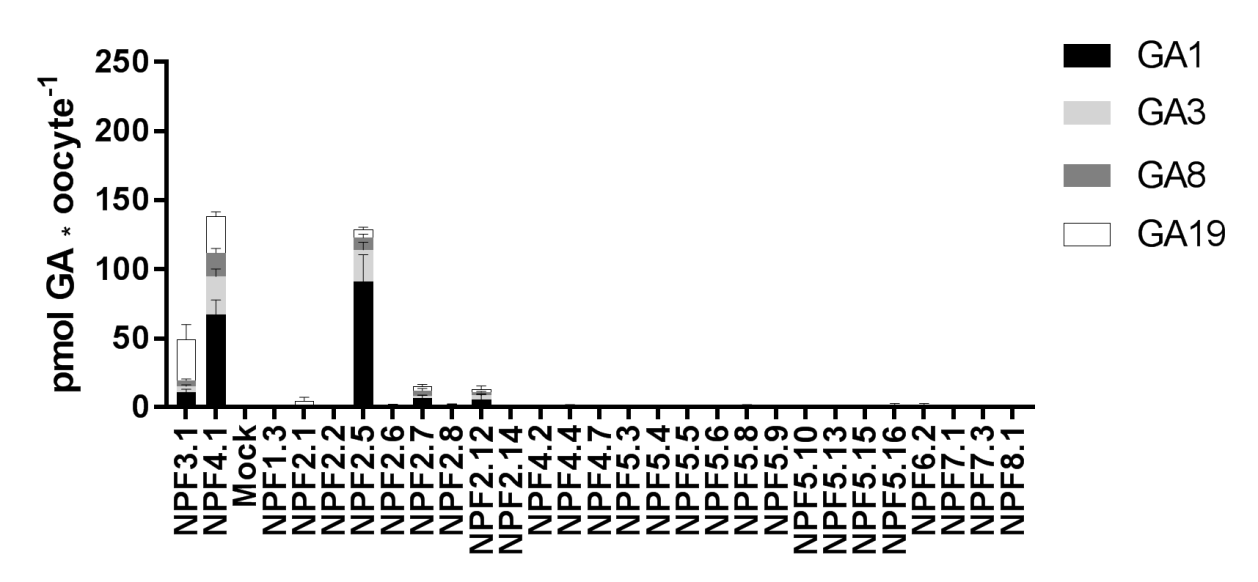

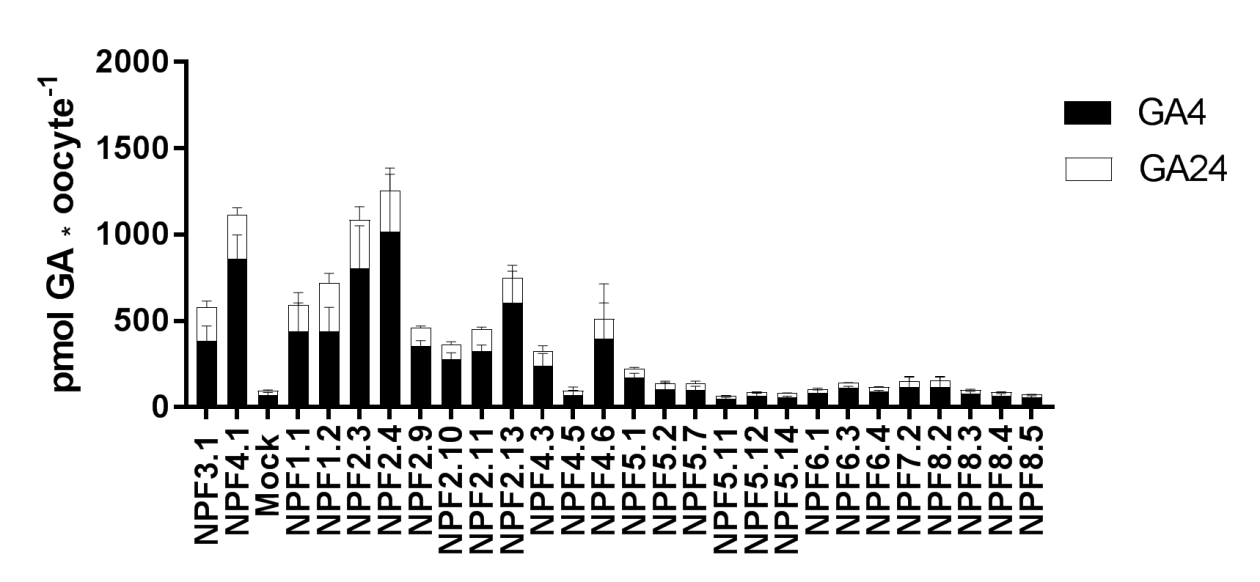

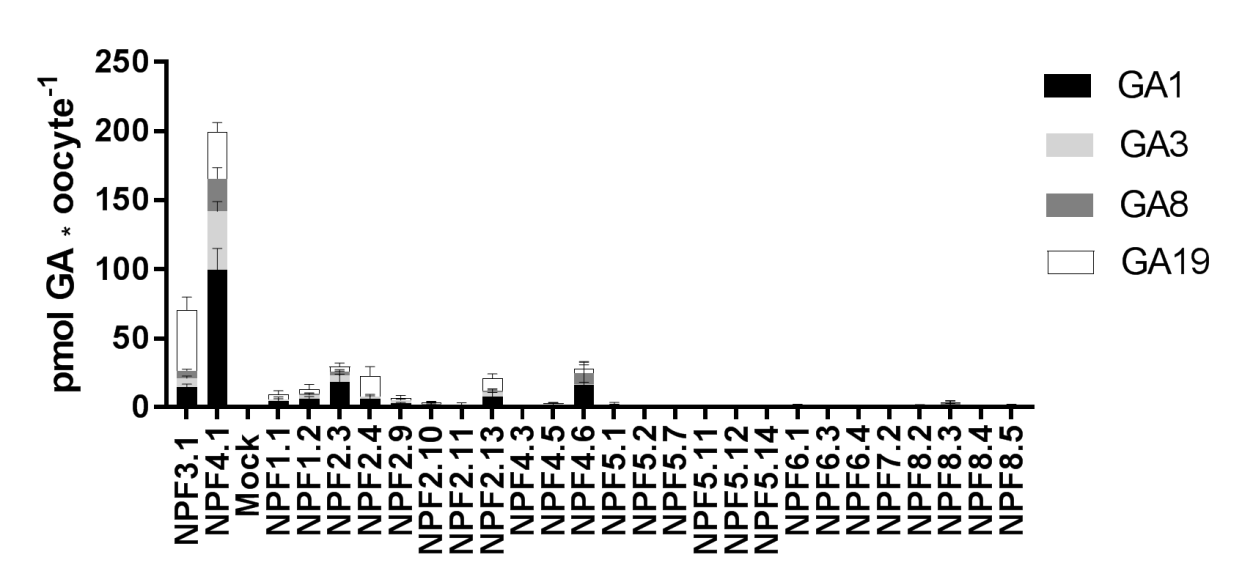


**Supplementary Figure 4.** Un-normalized data of first and second half of the quantitative screen. Both assays included NPF3.1, NPF4.1 and Mock in order to normalize. Oocytes (n = 5-6) were exposed to a mix of 50 µM GA1, 50 µM GA3, 100 µM GA4, 50 µM GA8, 50 µM GA19 and 50 µM GA24 in pH kulori 5.5 for 1h. Oocyte GA content was analyzed using LC-MS. For ease of visualization, data is divided to show accumulation of either membrane permeable or membrane non-permeable GAs.
