# Supplementary material for: An Optimized Screen Reduces the Number of GA Transporters and Provides Insights Into Nitrate Transporter 1/Peptide Transporter Family Substrate Determinants
Source: Front Plant Sci. 2019 Oct 3;10:1106. doi: 10.3389/fpls.2019.01106 (PMC6785635; doi:10.3389/fpls.2019.01106)
Supplement: Supplementary file 5 [file Table_5.docx]

Supplementary Material


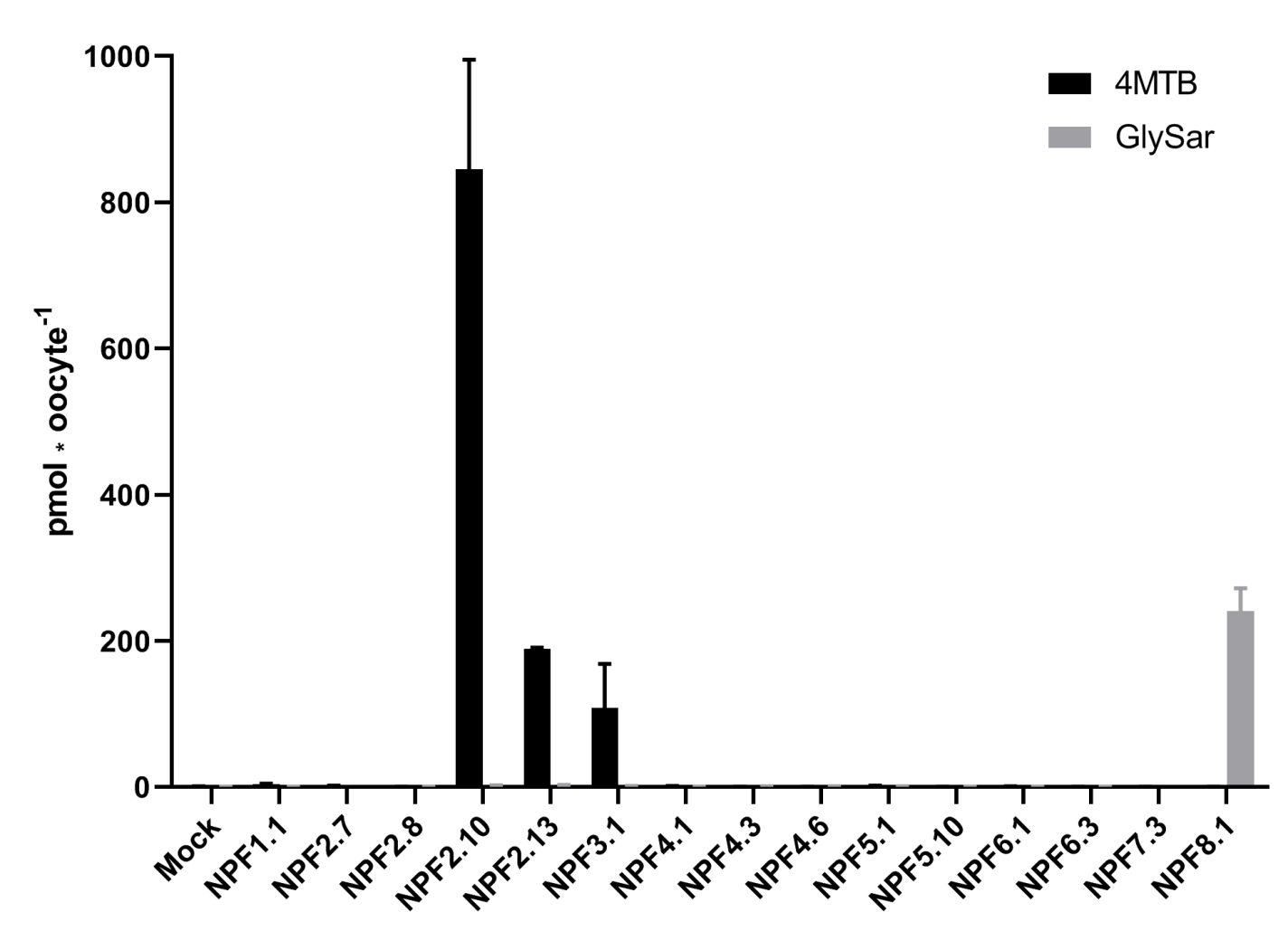


**Supplementary Figure 5.** Aliphatic glucosinolate and peptides transport are confined to branches in the NPF family. Oocytes (n = 5) expressing chosen Arabidopsis NPF members were subjected to a mix of 500 µM 4-methylthio-3-butenyl aliphatic glucosinolate and 500 µM un-metabolizable glycyl-sarcosine dipeptide in MES based kulori pH 5 for 1 h and analyzed on LC-MS/MS.
