# Supplementary material for: An Optimized Screen Reduces the Number of GA Transporters and Provides Insights Into Nitrate Transporter 1/Peptide Transporter Family Substrate Determinants
Source: Front Plant Sci. 2019 Oct 3;10:1106. doi: 10.3389/fpls.2019.01106 (PMC6785635; doi:10.3389/fpls.2019.01106)
Supplement: Supplementary file 6 [file Table_6.docx]

Supplementary Material


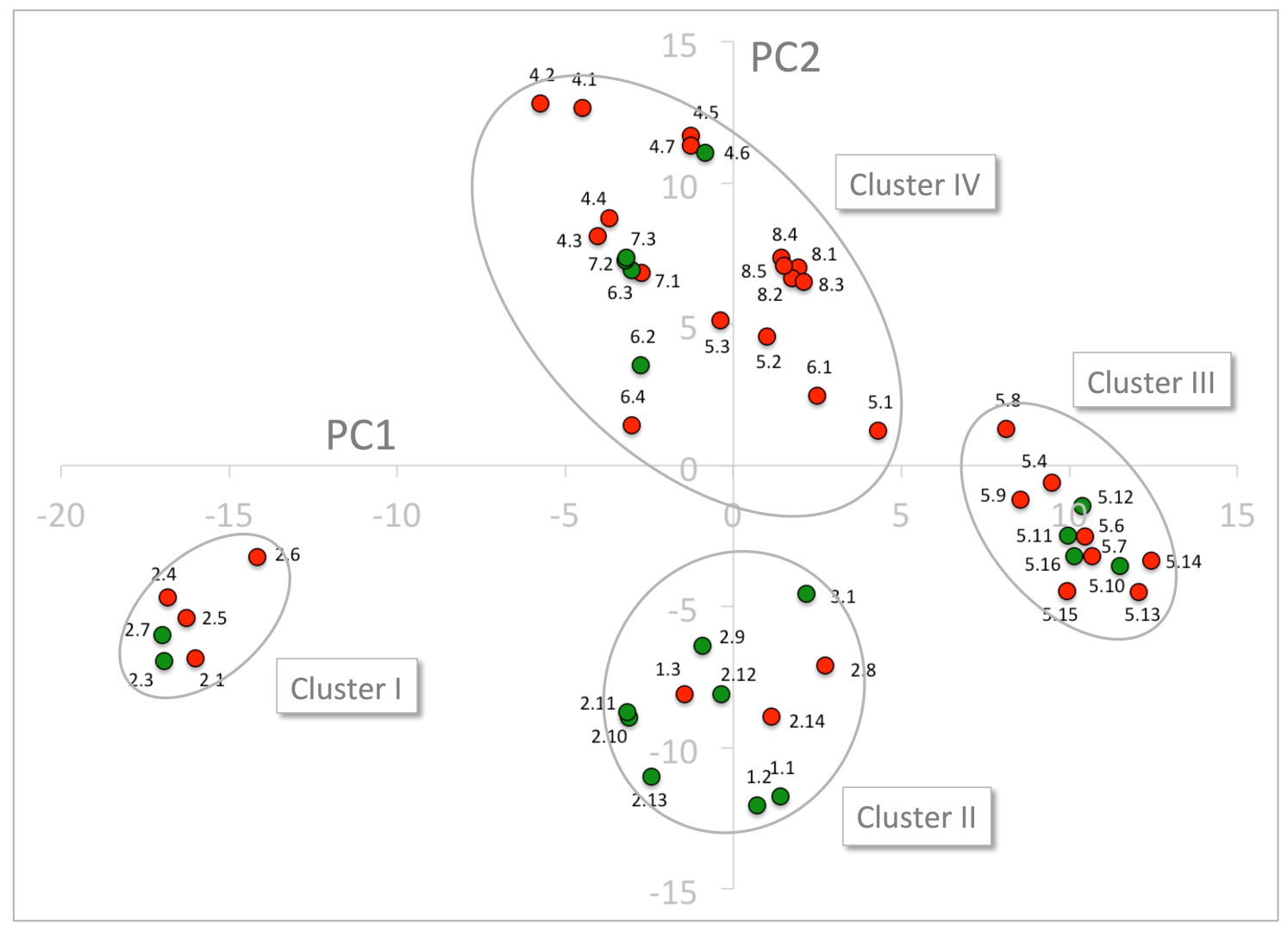


**Supplementary Figure 6.** Projection of nitrate transporting NPFs onto Principal Component Analysis of the 51 NPF sequences expressed by z-scales of the 51 cavity residues. Nitrate transporting transporters are shown as green dots and non-nitrate transporting transporters as red dots. The four clusters are marked by ellipses. PC1 and PC2 refer to the first and second principal components, respectively.
