# Supplementary material for: An Optimized Screen Reduces the Number of GA Transporters and Provides Insights Into Nitrate Transporter 1/Peptide Transporter Family Substrate Determinants
Source: Front Plant Sci. 2019 Oct 3;10:1106. doi: 10.3389/fpls.2019.01106 (PMC6785635; doi:10.3389/fpls.2019.01106)
Supplement: Supplementary file 7 [file Table_7.docx]

Supplementary Material


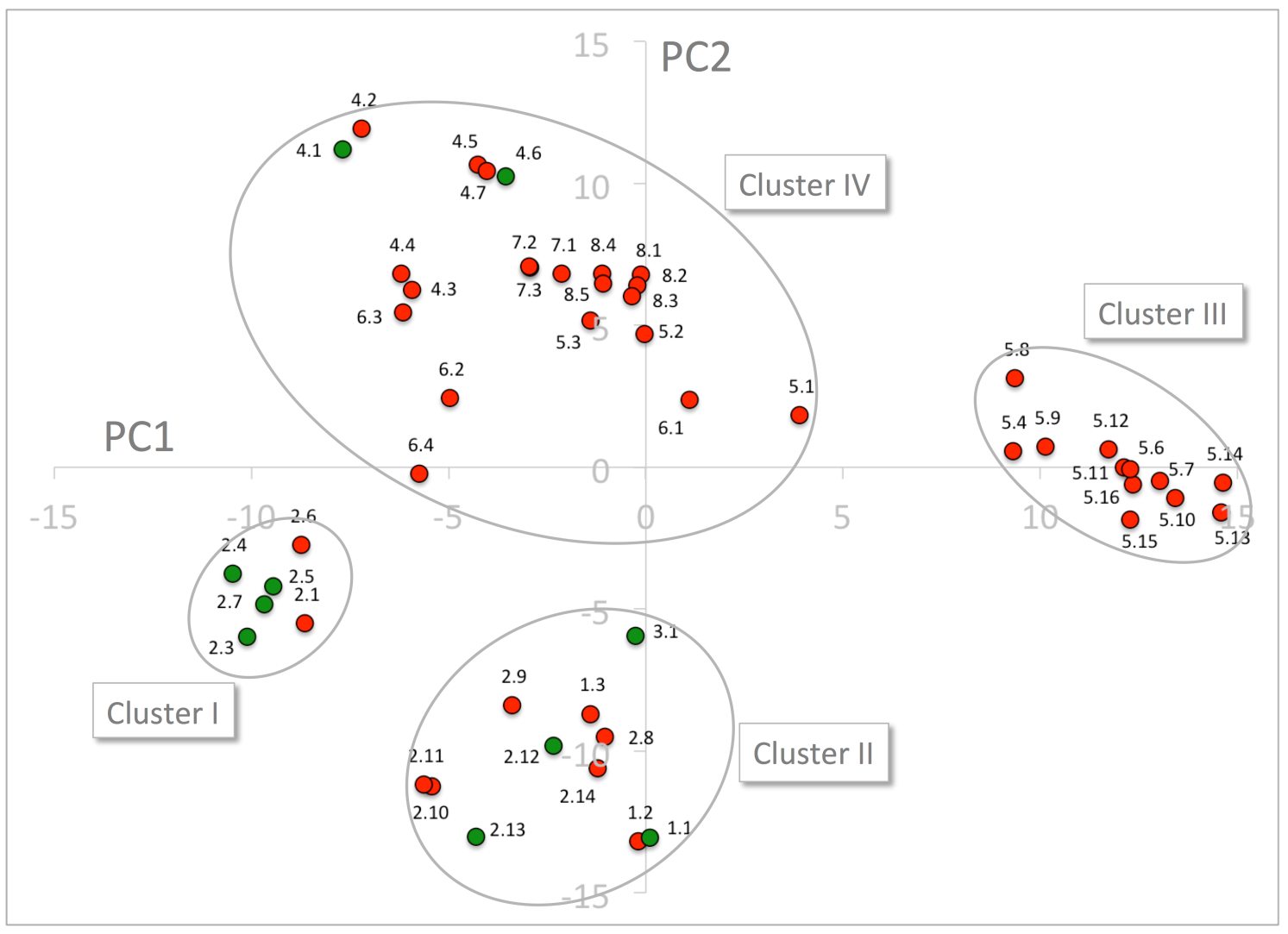


**Supplementary Figure 7.** Principal Component Analysis of the 51 NPF sequences expressed by z-scales of the 48 cavity residues that excludes the ExxE[K/R] motif. GA transporting and GA non-transporting transporters are shown as green and red dots, respectively. The four clusters are marked by ellipses. PC1 and PC2 refer to the first and second principal components, respectively.
