# Supplementary material for: An Optimized Screen Reduces the Number of GA Transporters and Provides Insights Into Nitrate Transporter 1/Peptide Transporter Family Substrate Determinants
Source: Front Plant Sci. 2019 Oct 3;10:1106. doi: 10.3389/fpls.2019.01106 (PMC6785635; doi:10.3389/fpls.2019.01106)
Supplement: Supplementary file 8 [file Table_8.docx]

Supplementary Material

| **Gene** | **Substrates** | **Non-substrate GAs** | **Ref** |
| --- | --- | --- | --- |
| AtNPF1.1 | GA1, GA3, GA4, ABA, JA-Ile  Nitrate | -  - | Chiba et al. 2015  Hsu et al. 2013 |
| AtNPF1.2 | GA1, GA3, GA4, JA-Ile  Nitrate | -  - | Chiba et al. 2015  Hsu et al. 2013. |
| AtNPF2.3 | GA1, GA3, (GA4)  Nitrate | -  - | Chiba et al. 2015  Taochy et al. 2015 |
| AtNPF2.4 | GA1, GA3, (GA4)  Chloride | -  - | Chiba et al. 2015  Li et al. 2016^1^ |
| AtNPF2.5 | GA1, GA3, (GA4), ABA  GA3  Chloride | -  -  - | Chiba et al. 2015  Kanno et al. 2016  Li et al. 2016^2^ |
| AtNPF2.6 | (GA1, GA4, JA-Ile) | GA3 | Chiba et al. 2015 |
| AtNPF2.7 | GA1, GA3  Nitrate | GA4 | Chiba et al. 2015  Segonzac et al. 2007 |
| AtNPF2.10 | **GA1, GA3**  **GA3,** ABA, JA, JA-Ile, TA  **GA3**  **-**  4MTB  I3M | GA4  **GA1,** GA4, GA8, GA9, GA20  -  **GA3,** GA4  -  - | Chiba et al. 2015  Saito et al. 2015  Kanno et al. 2016  Tal et al. 2016  Nour-Eldin et al. 2012  Jorgensen et al. 2017 |
| AtNPF2.11 | GA3  GA3  4MTB  I3M | -  -  -  - | Saito et al. 2015  Tal et al. 2016  Nour-Eldin et al. 2012  Jorgensen et al. 2017 |
| AtNPF2.12 | GA1, GA3  Nitrate | GA4  - | Chiba et al. 2015  Almagro et al. 2008 |
| AtNPF2.13 | GA1, GA3, (GA4)  Nitrate  4MTB  I3M | -  -  -  - | Chiba et al. 2015  Fan et al. 2009  Nour-Eldin et al. 2012  Jorgensen et al. 2017 |
| AtNPF3.1 | GA1, GA3  GA1, GA3, **GA4**, GA8, **GA20**  GA1, GA3, **GA4**, GA8, GA19  -  Nitrate, Nitrite | **GA4**  GA9, GA12  GA9, GA12, GA15, **GA20**, GA24, GA44, GA53  - | Chiba et al. 2015  Tal et al. 2016  David et al. 2016  David et al. 2016  Pike et al. 2014 |
| AtNPF4.1 | GA3, ABA, JA  GA1, GA3, GA4  GA3  GA3, GA4, GA8, GA20 | -  -  -  GA9, GA12 | Kanno et al. 2012  Chiba et al. 2015  Saito et al. 2015  Tal et al. 2016 |
| AtNPF4.2 | GA1, GA3, (GA4)  ABA | -  - | Chiba et al. 2015  Kanno et al. 2012 |
| AtNPF4.5 | (GA1, GA3), ABA  ABA | -  - | Chiba et al. 2015  Kanno et al. 2012 |
| AtNPF4.6 | (GA1, GA3), ABA  ABA  Nitrate | -  -  - | Chiba et al. 2015  Kanno et al. 2012  Huang et al. 1999 |
| AtNPF5.1 | GA1, GA3, (GA4) | - | Chiba et al. 2015 |
| AtNPF5.2 | GA1, GA3, (GA4), ABA  Peptides | -  - | Chiba et al. 2015  Karim et al. 2007 |
| AtNPF5.3 | (GA1, GA3, ABA) | GA4 | Chiba et al. 2015 |
| AtNPF5.6 | (GA1, GA3, GA4) | - | Chiba et al. 2015 |
| AtNPF5.7 | GA1, **GA3, GA4**, JA-Ile  **-** | -  **GA3, GA4** | Chiba et al. 2015  Tal et al. 2016 |
| AtNPF5.8 | (GA1, GA4) | GA3 | Chiba et al. 2015 |
| AtNPF5.9 | (GA1, GA4) | GA3 | Chiba et al. 2015 |
| AtNPF6.3 | (GA1, GA4)  Nitrate  IAA | GA3  -  - | Chiba et al. 2015  Tsay et al. 1993  Krouk et al. 2010 |
| AtNPF8.2 | (GA1, GA4, ABA), JA-Ile  Peptides  Peptides | GA3  -  - | Chiba et al. 2015  Komarova et al. 2008  Hammes et al. 2010 |
| AtSWEET13 | GA1, GA3, GA4, (GA9), GA12, GA15, GA19, GA20 GA24, GA44, GA53  Sucrose | GA8  -  - | Kanno et al. 2016  Kanno et al. 2016  Chen et al. 2012 |
| AtSWEET14 | GA1, GA3, GA4, GA8, GA9, GA12, GA15, GA19, GA20 GA24, GA44, GA53  Sucrose | -  -  - | Kanno et al. 2016  Kanno et al. 2016  Kanno et al. 2016  Chen et al. 2012 |

**Supplementary Table 1.** Summary of substrates of published GA transporters. Lack of consensus between GA transport assays is highlighted in **Bold**. Data presented in parentheses are regarded as positive transport events, but uncertainty exists. (Tsay, Schroeder et al. 1993, Huang, Liu et al. 1999, Karim, Holmstrom et al. 2007, Segonzac, Boyer et al. 2007, Almagro, Lin et al. 2008, Komarova, Thor et al. 2008, Fan, Lin et al. 2009, Hammes, Meier et al. 2010, Krouk, Lacombe et al. 2010, Chen, Qu et al. 2012, Kanno, Hanada et al. 2012, Nour-Eldin, Andersen et al. 2012, Hsu and Tsay 2013, Pike, Gao et al. 2014, Chiba, Shimizu et al. 2015, Saito, Oikawa et al. 2015, Taochy, Gaillard et al. 2015, David, Berquin et al. 2016, Kanno, Oikawa et al. 2016, Li, Byrt et al. 2016, Li, Qiu et al. 2016, Tal, Zhang et al. 2016, Jørgensen, Xu et al. 2017)

Almagro, A., S. H. Lin and Y. F. Tsay (2008). "Characterization of the Arabidopsis nitrate transporter NRT1.6 reveals a role of nitrate in early embryo development." Plant Cell **20**(12): 3289-3299.

Chen, L. Q., X. Q. Qu, B. H. Hou, D. Sosso, S. Osorio, A. R. Fernie and W. B. Frommer (2012). "Sucrose efflux mediated by SWEET proteins as a key step for phloem transport." Science **335**(6065): 207-211.

Chiba, Y., T. Shimizu, S. Miyakawa, Y. Kanno, T. Koshiba, Y. Kamiya and M. Seo (2015). "Identification of Arabidopsis thaliana NRT1/PTR FAMILY (NPF) proteins capable of transporting plant hormones." J Plant Res **128**(4): 679-686.

David, L. C., P. Berquin, Y. Kanno, M. Seo, F. Daniel-Vedele and S. Ferrario-Méry (2016). "N availability modulates the role of NPF3.1, a gibberellin transporter, in GA-mediated phenotypes in Arabidopsis." Planta: 1-14.

Fan, S.-C., C.-S. Lin, P.-K. Hsu, S.-H. Lin and Y.-F. Tsay (2009). "The Arabidopsis Nitrate Transporter NRT1.7, Expressed in Phloem, Is Responsible for Source-to-Sink Remobilization of Nitrate." The Plant Cell **21**(9): 2750-2761.

Hammes, U. Z., S. Meier, D. Dietrich, J. M. Ward and D. Rentsch (2010). "Functional properties of the Arabidopsis peptide transporters AtPTR1 and AtPTR5." J Biol Chem **285**(51): 39710-39717.

Hsu, P.-K. and Y.-F. Tsay (2013). "Two Phloem Nitrate Transporters, NRT1.11 and NRT1.12, Are Important for Redistributing Xylem-Borne Nitrate to Enhance Plant Growth." Plant Physiology **163**(2): 844-856.

Huang, N. C., K. H. Liu, H. J. Lo and Y. F. Tsay (1999). "Cloning and functional characterization of an Arabidopsis nitrate transporter gene that encodes a constitutive component of low-affinity uptake." Plant Cell **11**(8): 1381-1392.

Jørgensen, M. E., D. Xu, C. Crocoll, D. Ramírez, M. S. Motawia, C. E. Olsen, H. H. Nour-Eldin and B. A. Halkier (2017). "Origin and evolution of transporter substrate specificity within the NPF family." eLife **6**: e19466.

Kanno, Y., A. Hanada, Y. Chiba, T. Ichikawa, M. Nakazawa, M. Matsui, T. Koshiba, Y. Kamiya and M. Seo (2012). "Identification of an abscisic acid transporter by functional screening using the receptor complex as a sensor." Proc Natl Acad Sci U S A **109**(24): 9653-9658.

Kanno, Y., T. Oikawa, Y. Chiba, Y. Ishimaru, T. Shimizu, N. Sano, T. Koshiba, Y. Kamiya, M. Ueda and M. Seo (2016). "AtSWEET13 and AtSWEET14 regulate gibberellin-mediated physiological processes." Nature Communications **7**: 13245.

Karim, S., K. O. Holmstrom, A. Mandal, P. Dahl, S. Hohmann, G. Brader, E. T. Palva and M. Pirhonen (2007). "AtPTR3, a wound-induced peptide transporter needed for defence against virulent bacterial pathogens in Arabidopsis." Planta **225**(6): 1431-1445.

Komarova, N. Y., K. Thor, A. Gubler, S. Meier, D. Dietrich, A. Weichert, M. Suter Grotemeyer, M. Tegeder and D. Rentsch (2008). "AtPTR1 and AtPTR5 transport dipeptides in planta." Plant Physiol **148**(2): 856-869.

Krouk, G., B. Lacombe, A. Bielach, F. Perrine-Walker, K. Malinska, E. Mounier, K. Hoyerova, P. Tillard, S. Leon, K. Ljung, E. Zazimalova, E. Benkova, P. Nacry and A. Gojon (2010). "Nitrate-regulated auxin transport by NRT1.1 defines a mechanism for nutrient sensing in plants." Dev Cell **18**(6): 927-937.

Li, B., C. Byrt, J. Qiu, U. Baumann, M. Hrmova, A. Evrard, A. A. T. Johnson, K. D. Birnbaum, G. M. Mayo, D. Jha, S. W. Henderson, M. Tester, M. Gilliham and S. J. Roy (2016). "Identification of a Stelar-Localized Transport Protein That Facilitates Root-to-Shoot Transfer of Chloride in Arabidopsis." Plant Physiology **170**(2): 1014-1029.

Li, B., J. Qiu, M. Jayakannan, B. Xu, Y. Li, G. M. Mayo, M. Tester, M. Gilliham and S. J. Roy (2016). "AtNPF2.5 Modulates Chloride (Cl(-)) Efflux from Roots of Arabidopsis thaliana." Front Plant Sci **7**: 2013.

Nour-Eldin, H. H., T. G. Andersen, M. Burow, S. R. Madsen, M. E. Jørgensen, C. E. Olsen, I. Dreyer, R. Hedrich, D. Geiger and B. A. Halkier (2012). "NRT/PTR transporters are essential for translocation of glucosinolate defence compounds to seeds." Nature **488**(7412): 531-534.

Pike, S., F. Gao, M. J. Kim, S. H. Kim, D. P. Schachtman and W. Gassmann (2014). "Members of the NPF3 transporter subfamily encode pathogen-inducible nitrate/nitrite transporters in grapevine and Arabidopsis." Plant Cell Physiol **55**(1): 162-170.

Saito, H., T. Oikawa, S. Hamamoto, Y. Ishimaru, M. Kanamori-Sato, Y. Sasaki-Sekimoto, T. Utsumi, J. Chen, Y. Kanno, S. Masuda, Y. Kamiya, M. Seo, N. Uozumi, M. Ueda and H. Ohta (2015). "The jasmonate-responsive GTR1 transporter is required for gibberellin-mediated stamen development in Arabidopsis." Nat Commun **6**.

Segonzac, C., J. C. Boyer, E. Ipotesi, W. Szponarski, P. Tillard, B. Touraine, N. Sommerer, M. Rossignol and R. Gibrat (2007). "Nitrate efflux at the root plasma membrane: identification of an Arabidopsis excretion transporter." Plant Cell **19**(11): 3760-3777.

Tal, I., Y. Zhang, M. E. Jøgensen, O. Pisanty, I. C. R. Barbosa, M. Zourelidou, T. Regnault, C. Crocoll, C. Erik Olsen, R. Weinstain, C. Schwechheimer, B. A. Halkier, H. H. Nour-Eldin, M. Estelle and E. Shani (2016). "The Arabidopsis NPF3 protein is a GA transporter." Nat Commun **7**.

Taochy, C., I. Gaillard, E. Ipotesi, R. Oomen, N. Leonhardt, S. Zimmermann, J. B. Peltier, W. Szponarski, T. Simonneau, H. Sentenac, R. Gibrat and J. C. Boyer (2015). "The Arabidopsis root stele transporter NPF2.3 contributes to nitrate translocation to shoots under salt stress." Plant J **83**(3): 466-479.

Tsay, Y.-F., J. I. Schroeder, K. A. Feldmann and N. M. Crawford (1993). "The herbicide sensitivity gene CHL1 of arabidopsis encodes a nitrate-inducible nitrate transporter." Cell **72**(5): 705-713.
