# Supplementary material for: An Optimized Screen Reduces the Number of GA Transporters and Provides Insights Into Nitrate Transporter 1/Peptide Transporter Family Substrate Determinants
Source: Front Plant Sci. 2019 Oct 3;10:1106. doi: 10.3389/fpls.2019.01106 (PMC6785635; doi:10.3389/fpls.2019.01106)
Supplement: Supplementary file 9 [file Table_9.docx]

Supplementary Material

| Gene | AGI | Direction | Primer sequence | CDS Origin |
| --- | --- | --- | --- | --- |
| NPF1.1 | At3g16180 | Forward  Reverse | GGCTTAAUATGGAGAACCCTCCCAATGA  GGTTTAAUTTAATTGGTTTTAACAACTGGACTTAGATCTTC | Courtesy of Wolf B. Frommer |
| NPF1.2 | At1g52190 | - | - | uNCDF |
| NPF1.3 | At5g11570 | - | - | uNCDF |
| NPF2.1 | At3g45720 | Forward  Reverse | GGCTTAAUATGGCTGGTTTAGTATTATCTA  GGTTTAAUTTAGTTTGTAACATCTTTAGGA | Cloned from cDNA |
| NPF2.2 | At3g45690 | - | - | uNCDF |
| NPF2.3 | At3g45680 | - | - | uNCDF |
| NPF2.4 | At3g45700 | Forward  Reverse | GGCTTAAUATGGCTAATTCAGACTCTGGTGACAAAGAA  GGTTTAAUCTAGTTTGTAACATCTTTAAGATCTTGTTCATGATC | Courtesy of Wolf B. Frommer |
| NPF2.5 | At3g45710 | Forward  Reverse | GGCTTAAUATGGCTGATTCAAAATCTGGTGAC  GGTTTAAUCTAGGTTTTAACATCTTTAGGATCTTGTTCATG | Cloned from cDNA |
| NPF2.6 | At3g45660 | - | - | uNCDF |
| NPF2.7 | At3g45650 | Forward  Reverse | GGCTTAAUATGGCTAGTTCAGTTACTG  GGTTTAAUTCAGTGAGAGACATTTGC | Cloned from cDNA |
| NPF2.8 | At5g28470 | - | - | uNCDF |
| NPF2.9 | At1g18880 | Forward  Reverse | GGCTTAAUATGGAGGTTGAGAAGACAG  GGTTTAAUTTACACTGACACCTTATCAAAC | Courtesy of Wolf B. Frommer |
| NPF2.10 | At3g47960 | Forward  Reverse | GGCTTAAUATGAAGAGCAGAGTCATT  GGTTTAAUTCAGACAGAGTTCTTGTC | RIKEN BRC |
| NPF2.11 | At5g62680 | Forward  Reverse | GGCTTAAUATGGAGAGAAAGCCTCTTGAAC  GGTTTAAUTCAGGCAACGTTCTTGTCTTG | Nour Eldin et al. 2012) |
| NPF2.12 | At1g27080 | Forward  Reverse | GGCTTAAUATGGGAGTTGTTGAGAATCGG  GGTTTAAUCTAACTTGGAGATTGATCGTGTCTC | Courtesy of Eilon Shani |
| NPF2.13 | At1g69870 | Forward  Reverse | GGCTTAAUATGGTTTTGGAGGATAGAAAGGAC  GGTTTAAUTCATTTCATCGATTTCTTCGAAGTCATCTC | Nour Eldin et al. 2012) |
| NPF2.14 | At1g69860 | Forward  Reverse | GGCTTAAUATGGACAATGAGAAAGGGACAAG  GGTTTAAUTTATTGTTCATTACTATAGTTTCTGTAACGATACC | (Nour Eldin et al. 2012) |
| NPF3.1 | At1g68570 | Forward  Reverse | GGCTTAAUATGGAGGAGCAAAGCAAGAA  GGTTTAAUTCATTCATCAACTAAACTCCTA | RIKEN BRC |
| NPF4.1 | At3g25260 | Forward  Reverse | GGCTTAAUATGCAGATTGAGATGGAAGAG  GGTTTAAUCTAATATCTTTTCGCCCAG | Cloned from cDNA |
| NPF4.2 | At3g25280 | - | - | uNCDF |
| NPF4.3 | At1g59740 | Forward  Reverse | GGCTTAAUATGGCAGAGATAAACAAACAAAGCA  GGTTTAAUCTAAATGTTCTCATCACCCACAAC | RIKEN BRC |
| NPF4.4 | At1g33440 | - | - | uNCDF |
| NPF4.5 | At1g27040 | Forward  Reverse | GGCTTAAUATGGAAGTAGAAATGCATGGTGA  GGTTTAAUTCAACTTATTGAACCAGTTGAGATATAC | RIKEN BRC |
| NPF4.6 | At1g69850 | Forward  Reverse | GGCTTAAUATGGAAGTGGAAGAAGAGGTCTC  GGTTTAAUTTAGCTTCTTGAACCAGTTGATCTATAC | Courtesy of Wolf B. Frommer |
| NPF4.7 | At5g62730 | - | - | uNCDF |
| NPF5.1 | At2g40460 | Forward  Reverse | GGCTTAAUATGGAGGCTGCAAAAGTTTACAC  GGTTTAAUTTAGATACTAAGAGGAGATGTGTCTAAGGC | Courtesy of Wolf B. Frommer |
| NPF5.2 | At5g46050 | Forward  Reverse | GGCTTAAUATGACAGTAGAAGAGGTAGGAGAC  GGTTTAAUTTATTCAGTCTCTTTCATTTCCACCTC | RIKEN BRC |
| NPF5.3 | At5g46040 | Forward  Reverse | GGCTTAAUATGACAGTAGAAGAGGTAGG  GGTTTAAUTTACTCATTGTAGTTATCTACC | Cloned from cDNA |
| NPF5.4 | At3g54450 | - | - | uNCDF |
| NPF5.5 | At2g38100 | - | - | uNCDF |
| NPF5.6 | At2g37900 | - | - | uNCDF |
| NPF5.7 | At3g53960 | - | - | uNCDF |
| NPF5.8 | At5g14940 | - | - | uNCDF |
| NPF5.9 | At3g01350 | - | - | uNCDF |
| NPF5.10 | At1g22540 | Forward  Reverse | GGCTTAAUATGTCGATCTCCGGCGCT  GGTTTAAUTTAACTGGTGTCGAGCCTTT | Cloned from cDNA |
| NPF5.11 | At1g72130 | Forward  Reverse | GGCTTAAUATGGCTATCACCTACTCCTCC  GGTTTAAUTTAAAAGGTGTTTGATCTGCTGTAGACAT | RIKEN BRC |
| NPF5.12 | At1g72140 | Forward  Reverse | GGCTTAAUATGTCGACATCCATCGGCG  GGTTTAAUCTACTTTGGGCTGTTGTAGAGATAG | RIKEN BRC |
| NPF5.13 | At1g72125 | Forward  Reverse | GGCTTAAUATGACGACGACTTCCAAAAC  GGTTTAAUCTACACTACGTCCACCCG | Cloned from cDNA |
| NPF5.14 | At1g72120 | Forward  Reverse | GGCTTAAUATGACGACTACTTCAGAAATTTCTCT  GGTTTAAUCTACACTCGATCCACTCGACG | RIKEN BRC |
| NPF5.15 | At1g22570 | Forward  Reverse | GGCTTAAUATGAAGATACCAGAGGAAGAAGTTGC  GGTTTAAUTTAGACTTGGTCTAGCCGACG | Cloned from cDNA |
| NPF5.16 | At1g22550 | Forward  Reverse | G GGCTTAAUATGGCGATAGCCGAAGAAGAA  GGTTTAAUTTAGACTTGATCTACACGGCG | Courtesy of Wolf B. Frommer |
| NPF6.1 | At5g13400 | Forward  Reverse | GGCTTAAUATGGTTGCTTCTGAGATTAAATCCC  GGTTTAAUTTAAAGGACAGCACTACTCTTGTCTTCC | Courtesy of Wolf B. Frommer |
| NPF6.2 | At2g26690 | Forward  Reverse | GGCTTAAUATGGAGAGCAAAGGGAGTTGG  GGTTTAAUTCAGCAGTCTTCAACTGAAAATCC | RIKEN BRC |
| NPF6.3 | At1g12110 | Forward  Reverse | GGCTTAAUATGTCTCTTCCTGAAACTAAATCTG  GGTTTAAUATGACCCATTGGAATACTCG | Courtesy of Wolf B. Frommer |
| NPF6.4 | At3g21670 | Forward  Reverse | GGCTTAAUATGGTTCATGTGTCATCATCTCATG  GGTTTAAUTCAAGGAATGTCCTTAAGCTCAA | Courtesy of Wolf B. Frommer |
| NPF7.1 | At5g19640 | Forward  Reverse | GGCTTAAUATGGCCGCTATGGATCCG  GGTTTAAUAGTTTGAACAAGGTTGAGTCTTT | Cloned from cDNA |
| NPF7.2 | At4g21680 | Forward  Reverse | GGCTTAAUATGGATCAAAAAGTTAGACAGT  GGTTTAAUTCAGACTTCCTCCTCTTCAGTTA | Courtesy of Wolf B. Frommer |
| NPF7.3 | At1g32450 | Forward  Reverse | GGCTTAAUATGTCTTGCCTAGAGATTTAT  GGTTTAAUTTAGACTTTAGAATCCTTCTCT | Courtesy of Wolf B. Frommer |
| NPF8.1 | At3g54140 | Forward  Reverse | GGCTTAAUATGGAAGAAAAAGATGTGTATACGC  GGTTTAAUTCAATGTGCTCGACCAACAGC | Courtesy of Wolf B. Frommer |
| NPF8.2 | At5g01180 | Forward  Reverse | GGCTTAAUATGGAAGATGACAAGGATATATACACAAA  GGTTTAAUTCAAAGCGCATGCCCGGT | Courtesy of Wolf B. Frommer |
| NPF8.3 | At2g02040 | - | - | uNCDF |
| NPF8.4 | At2g02020 | - | - | uNCDF |
| NPF8.5 | At1g62200 | Forward  Reverse | GGCTTAAUATGGTGAATTCGAATGAAGAAGACG  GGTTTAAUTTACAAAGCCTTCTTCTTTGTGTGC | Courtesy of Wolf B. Frommer |

**Supplementary Table 2.** List of primers and CDS origin. Uracil containing Non-clonal DNA fragments (uNCDF) .
