# Supplementary material for: An Optimized Screen Reduces the Number of GA Transporters and Provides Insights Into Nitrate Transporter 1/Peptide Transporter Family Substrate Determinants
Source: Front Plant Sci. 2019 Oct 3;10:1106. doi: 10.3389/fpls.2019.01106 (PMC6785635; doi:10.3389/fpls.2019.01106)
Supplement: Supplementary file 10 [file Table_10.docx]

Supplementary Material

| **Analyte** | **RT [min]** | **Q1** **[*m/z*]** | **Q3 [*m/z*]** | **CE [eV]** |
| --- | --- | --- | --- | --- |
| GA1 [M-H]^-^ | 2.29 | 347.16 | 259.1^Q^ | 16 |
|  |  | 347.16 | 145.2 | 28 |
| GA3 [M-H]^-^ | 2.27 | 345.2 | 243.1^Q^ | 25 |
|  |  | 345.2 | 239.1 | 12 |
|  |  | 345.2 | 221.1 | 21 |
| GA4 [M-H]^-^ | 2.62 | 331.1 | 243.0^Q^ | 16 |
|  |  | 331.1 | 225.1 | 15 |
|  |  | 331.1 | 213.0 | 30 |
|  |  | 331.1 | 287,1 | 19 |
| GA7 [M-H]^-^ | 2.64 | 329.15 | 223.1^Q^ | 16 |
|  |  | 329.15 | 211.1 | 21 |
|  |  | 329.15 | 155.0 | 27 |
| GA8 [M-H]^-^ | 2.05 | 363.2 | 275.1^Q^ | 15 |
|  |  | 363.2 | 257.1 | 15 |
|  |  | 363.2 | 119.2 | 23 |
|  |  | 363.2 | 319,1 | 11 |
| GA9 [M-H]^-^ | 2.82 | 315.2 | 271.1^Q^ | 16 |
|  |  | 315.2 | 253.1 | 23 |
| GA12 [M-H]^-^ | 2.89 | 331.2 | 313.1^Q^ | 24 |
|  |  | 331.2 | 287.1 | 22 |
| GA19 [M-H]^-^ | 2.38 | 331.2 | 273.0^Q^ | 24 |
|  |  | 331.2 | 203.0 | 29 |
|  |  | 331.2 | 317.0 | 22 |
|  |  | 331.2 | 229.0 | 29 |
| GA24 [M-H]^-^ | 2.61 | 345.1 | 257.0^Q^ | 25 |
|  |  | 345.1 | 213.0 | 25 |
|  |  | 345.1 | 301.0 | 16 |
| 4-methylthio-3-butenyl [M-H]^-^ | 2.27 | 420.0 | 97.0^Q^ | 23 |
|  |  | 420.0 | 259.0 | 23 |
|  |  | 420.0 | 75.0 | 30 |
| Glycyl-sarcosine [M+H]^+^ | 0.58 | 147.1 | 90.2^Q^ | -8 |
|  |  | 147.1 | 44.4 | -17 |
| JA [M-H]^-^ | 2.45 | 209.1 | 59.3^Q^ | 11 |
| JA-Ile [M-H]^-^ | 2.49 | 322.0 | 130.1^Q^ | 17 |
| ABA [M-H]^-^ | 2.34 | 263.0 | 153.1^Q^ | 7 |
|  |  | 263.0 | 151.0 | 7 |
| OPDA [M-H]^-^ | 2.79 | 291.0 | 165.1^Q^ | 17 |
|  |  | 291.0 | 273.1 | 13 |
| Sinigrin [M-H]^-^ (IS) | 1.60 | 358.0 | 97.0^Q^ | 22 |
|  |  | 358.0 | 79.0 | 30 |
|  |  | 358.0 | 259.0 | 20 |

**Supplementary Table 3.** Multiple reaction monitoring transitions for analyte detection by LC-MS/MS. ^Q^Quantifier ion used for quantification of the respective analyte. Other transitions were used for compound identification together with retention times compared to those of standards. IS = internal standard.
